# Correlated Response to Selection for Litter Size Residual Variability in Rabbits’ Body Condition

**DOI:** 10.3390/ani10122447

**Published:** 2020-12-21

**Authors:** Iván Agea, María de la Luz García, Agustín Blasco, Peter Massányi, Marcela Capcarová, María-José Argente

**Affiliations:** 1 Departamento de Tecnología Agroalimentaria, Escuela Politécnica Superior de Orihuela, Universidad Miguel Hernández de Elche, Ctra. de Beniel km 3.2, 03312 Orihuela, Spain; iagea@umh.es (I.A.); mariluz.garcia@umh.es (M.d.l.L.G.); 2 Institute for Animal Science and Technology, Universitat Politècnica de València, P.O. Box 22012, 46022 València, Spain; ablasco@dca.upv.es; 3 Department of Animal Physiology, Faculty of Biotechnology and Food Sciences, Slovak University of Agriculture, 949 76 Nitra, Slovakia; massanyip@gmail.com (P.M.); marcela.capcarova@uniag.sk (M.C.)

**Keywords:** body condition, fertility, litter size variability, rabbits, selection

## Abstract

**Simple Summary:**

Selection for decreasing litter size residual variance has been proposed as an indirect way to select for resilience. Resilience has been directly related to welfare. A good body condition and efficient body fat mobilization have been associated with an optimal level of animal welfare. Two rabbit lines have been divergently selected for litter size residual variability. The low line selected for decreasing litter size variance more efficiently managed the body fat from mating to weaning in the second productive cycle in females compared to the high line, which could be related to the lower culling rate reported previously in the low line. Therefore, body condition can be used as a useful biomarker of resilience.

**Abstract:**

A divergent selection experiment for residual variance of litter size at birth was carried out in rabbits during twelve generations. Residual variance of litter size was estimated as the within-doe variance of litter size after pre-correction for year and season as well as parity and lactation status effects. The aim of this work was to study the correlated response to selection for litter size residual variability in body condition from mating to weaning. Body condition is related directly to an animal’s fat deposits. Perirenal fat is the main fat deposit in rabbits. Individual body weight (IBW) and perirenal fat thickness (PFT) were used to measure body condition at second mating, delivery, 10 days after delivery, and weaning. Litter size of the first three parities was analyzed. Both lines decreased body condition between mating to delivery; however, the decrease in body condition at delivery was lower in the low line, despite this line having higher litter size at birth (+0.54 kits, *p* = 0.93). The increment of body condition between delivery and early lactation was slightly higher in the low line. On the other hand, body condition affected success of females’ receptivity and fertility at the third mating, e.g., receptive females showed a higher IBW and PFT than unreceptive ones (+129 g and +0.28 mm, respectively), and fertile females had a higher IBW and PFT than unfertile ones (+82 g and +0.28 mm, respectively). In conclusion, the does selected for reducing litter size variability showed a better deal with situations of high-energy demand, such as delivery and lactation, than those selected for increasing litter size variability, which would agree with the better health and welfare condition in the low line.

## 1. Introduction

Animal welfare is a priority in livestock production for ethical reasons and also because poor animal welfare is associated with low production, poor health, and larger culling rate [1]. Resilience is defined as the ability of an animal to maintain or quickly recover its performance in spite of environmental perturbations [2,3], thus it is directly related to welfare. The ability of an animal to efficiently mobilize its fat reserves can be essential for it to maintain, or quickly return to its production level. Body condition has been traditionally employed to measure the mobilization of fat reserves in livestock animals (Schröder and Staufenbiel [4] in cattle; Maes et al. [5] in pigs; Pascual et al. [6] in rabbits). Body condition has been commonly used as a welfare indicator, due to its relations with fertility success and prevention of diseases (Barletta et al. [7] in cattle; van Staaveren et al. [8] in pigs; Sánchez et al. [9] in rabbits). Therefore, body condition may be connected to resilience, and monitoring it may be useful in resilience assessments.

Recently, residual variance has been proposed as a measure of resilience [10,11]. A direct divergent selection experiment for residual variance in litter size has been performed successfully in rabbits at the Universidad Miguel Hernández de Elche [12]. The high and low lines showed a remarkable difference in residual variance of litter size (4.5% of the mean of the base population). There were also differences in sensitivity to stress and diseases, which lowered the culling rate in the low line [11]. In this regard, the more homogeneous line coped better with environmental stressors such as infections and acute stress than the heterogeneous line which showed higher resilience [11,13].

In an early experiment with the first generations of selection, García et al. [14] found that the low line had a favorable correlated response to selection in body condition and fat reserve mobilization at birth. The objective of this work was to study the correlated response to selection for litter size residual variability in the development of body condition from mating to weaning.

## 2. Materials and Methods

### 2.1. Ethics Statement

All experimental procedures were approved by the Miguel Hernández University of Elche Research Ethics Committee, according to Council Directives 98/58/EC and 2010/63/EU (reference number 2017/VSC/PEA/00212).

### 2.2. Experimental Animals

Animals came from the twelfth generation of a divergent selection experiment for residual variance of litter size (see more details in Blasco et al. [12]). A total of 121 females of the low line (homogeneous) and 124 females of the high line (heterogeneous) were used to estimate the response to selection and correlated responses in litter size at first, second and third parity, and correlated responses in individual body weight at 4 weeks and 9 weeks old. A subset of 100 primiparous females from the low line and 74 primiparous females from the high line were used to measure the development of body condition in the second reproductive cycle and to study the body condition effect on doe’s receptivity and fertility.

All animals were kept on a farm at the Miguel Hernández University of Elche (Spain). Rabbits were fed a standard commercial diet (17% crude protein, 16% fiber, 3.5% fat, Nutricun Elite Gra^®^, De Heus Nutrición Animal, La Coruña). Food and water were provided ad libitum. Females were housed in individual cages (37.5 cm × 33 cm × 90 cm) under a constant photoperiod of 16 h continuous light (8 h continuous darkness and controlled ventilation throughout the experiment). The experiment took place from December to August. They were first mated at 18 weeks of age and at 10 days after parturition thereafter. Gestation was checked by abdominal palpation 12 d after mating. Litters were not standardized and weaning was at 28 d after delivery.

### 2.3. Traits

Individual body weight at 4 weeks and 9 weeks old and litter size at birth were recorded. Residual variance of litter size was estimated for all females of the twelfth generation considering all parties, after pre-correcting litter size for the effects of year and season and parity and lactation status. Individual body weight and perirenal fat thickness were recorded at four different physiological stages: second mating, delivery, 10 days after delivery and weaning. Perirenal fat thickness was measured by ultrasound imaging to evaluate body fat reserves as described by Pascual et al. [15] using Toshiba NemioMX SSA-590 ultrasound equipment (Toshiba, Tokyo, Japan). Receptivity and fertility were recorder at third mating (i.e., 10 days after second delivery). Receptivity (acceptance or rejection of the male at mating) was defined as a binary trait as was fertility (pregnant or non-pregnant females at palpation).

### 2.4. Statistical Analysis

#### 2.4.1. Correlated Response to Selection for Residual Variance

Models included a different set of effects depending on the trait. The following models were used:

residual variance of litter size at birth had only the effect of line (two levels, high and low line); individual body weight at 4 weeks and 9 weeks old, litter size at first parity had the effects of line and season;

litter size at second and third parity had the effects of line, season and lactation status (two levels: lactating and non-lactating female at mating);

individual body weight and perirenal fat thickness had the effects of line-time (eight levels: low line at mating, high line at mating, low line at delivery, high line at delivery, low line 10 days after delivery, high line 10 days after delivery, low line at weaning, and high line at weaning), season, lactation status (two levels: lactating and non-lactating female when recording data) and the dam permanent effect.

All analyses were performed using Bayesian methodology [16]. Bounded uniform priors were used for all effects with the exception of the dam permanent effect, considered normally distributed with mean 0 and variance $\sigma_{p}^{2}$. Residuals were a priori normally distributed with mean 0 and variance $\sigma_{e}^{2}$ and uncorrelated with the dam effects. The priors for the variances were also bounded uniform. Features of the marginal posterior distributions for all unknowns were estimated using Gibbs sampling. The Rabbit program developed by the Institute for Animal Science and Technology (Valencia, Spain) was used for all procedures. We used a chain of 60,000 samples, with a burn-in period of 10,000. Only one out of every 10 samples were saved for inferences. Convergence was tested using the Z criterion of Geweke and Monte Carlo sampling errors were computed using time-series procedures. 

#### 2.4.2. Effect of Body Condition on Receptivity and Fertility

We analyzed the difference on body condition at third mating (i.e., at 10 days after second delivery) between receptive and non-receptive does, using a model with the effects of line, season, lactation status, and receptivity with two levels (acceptance or rejection of the male at first attempt). In order to study the difference on body condition at mating between fertile and unfertile does, we used a model with the effects of line, season, lactation status and fertility with two levels (pregnant or non-pregnant female at palpation).

A probit regression was performed to assess the effect of individual body weight and perirenal fat thickness at third mating on probability of successful receptivity and fertility using the former models. The probit procedure of the statistical package SAS was used for this analysis (SAS Institute, 2019, Cary, CA, USA).

## 3. Results

### 3.1. Correlated Response to Selection for Residual Variance

Table 1 shows the features of marginal posterior distributions of the differences between lines for litter size residual variance, litter size at first, second and third parity, and individual body weight at 4 weeks and 9 weeks old. The probability of these differences being greater than zero if D_L-H_ > 0 or lower than zero if D_L-H_ < 0 is shown. In a Bayesian context there are no significance levels; instead, we offer the actual probability of the differences. As the environmental effects are the same for both lines, the differences between lines (D_L-H_) are genetic differences, so they estimate the response and correlated responses to selection. The low line showed a lower litter size variability than the high line (–1.45 kits^2^, *p* =1.00), and a higher litter size in the first parities (+0.42 kits, *p* = 0.90 in first parity; +0.54 kits, *p* = 0.93 in second parity; +0.66 kits, *p* = 0.94 in third parity). The low line had similar body weight to the high line at 4 weeks and 9 weeks old.

**Figure 1:**
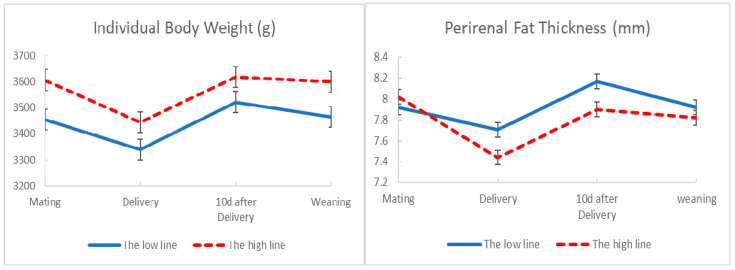
Development of individual body weight and perirenal fat thickness at second mating, delivery, 10 days after delivery and weaning in the low and high lines. The bars show standard deviation.

Figure 1 displays the development of body condition from second mating to weaning in the high and the low line. Individual body weight and perirenal fat thickness showed a reduction from mating to delivery in both lines. However, this reduction was lesser in the low line than in the high line. Both lines exhibited a recovery of body reserves from delivery to 10 days after delivery, but the increment was slightly higher in the low line. Body condition showed a decrease from 10 days after delivery to weaning, but the decrease was slightly higher in the low line. We notice that although number of kits at birth was higher in the low line than the high one (8.31 kits vs. 7.77 kits respectively, *p* = 0.93) perirenal fat thickness was higher in the low line than the high one in the critical moments of delivery (7.71 mm versus 7.44 mm, *p* = 0.99) and 10 days after delivery (8.17 mm versus 7.90 mm, *p* = 0.99).

### 3.2. Effect of Body Condition on Receptivity and Fertility

Table 2 shows that receptive females had higher individual body weight and perirenal fat thickness than unreceptive females (+129 g, *p* = 0.97 for body weight; +0.28 mm, *p* = 0.96 for perirenal fat thickness). Individual body weight and perirenal fat thickness were higher in fertile females compared to unfertile females (+82 g, *p* = 0.94 for body weight; +0.28 mm, *p* = 0.99 for perirenal fat thickness).

**Figure 2:**
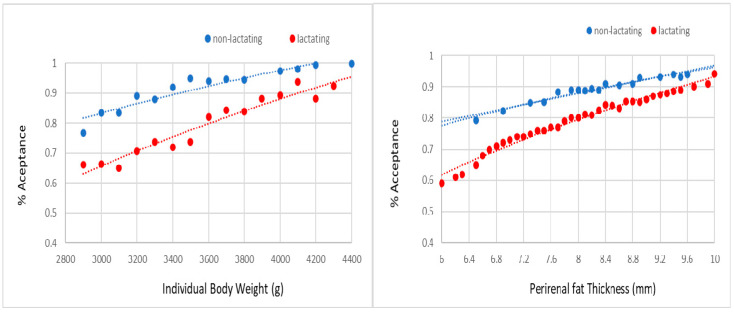
Probability of acceptance of the male at mating on individual body weight and perirenal fat thickness at 10 day after delivery (i.e., third mating in lactating and non-lactating does).

**Figure 3:**
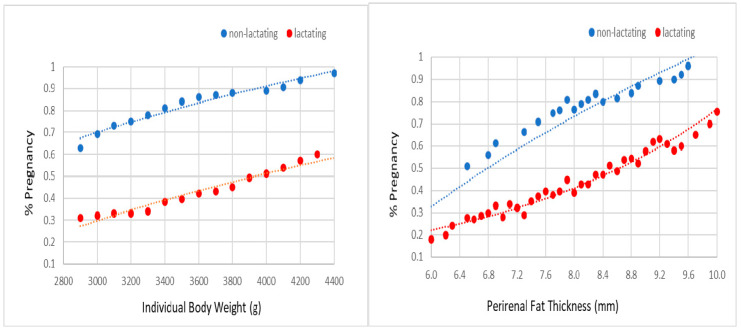
Probability of pregnancy at third gestation on individual body weight and perirenal fat thickness at 10 day after delivery (i.e., third mating, in lactating and non-lactating does).

The probabilities of acceptance of mating and pregnancy were not affected by line and season. However, non-lactating females always showed a higher probability for accepting the male and becoming pregnant than lactating females (Figure 2 and Figure 3). For a body weight between 2900 and 4400 g, the probability of acceptance of the male ranged from 75% to 100% in non-lactating does and from 65% to 95% in lactating does. For a perirenal fat thickness between 6.0 and 10.0 mm, the probability of acceptance of the male ranged from 80% to 95% in non-lactating does and from 60% to 95% in lactating does. For the same range of weights, the probability of pregnancy extended from 60% to 95% in non-lactating does and from 30% to 60% in lactating does. For the same range of perirenal fat thickness, the probability of pregnancy ranged from 50% to 95% in non-lactating does and from 20% to 80% in lactating does.

## 4. Discussion

### 4.1. Correlated Response to Selection for Residual Variance

We have found that, as in former generations [12], selection to reduce litter size residual variance produces females with more uniform litters. Uniformity in litter size and body weight has been related to immune response and resistance to diseases (see review [17]). Additionally, we have found that selection for reducing residual variance of litter size increases litter size without affecting the individual weight neither at birth nor at weaning [18].

In relation to development of body condition, we have observed that both lines decrease the body condition from mating to delivery. This is due to the negative energy balance during the last week of gestation, as a consequence of the growing fetuses and the decreasing feed intake in the mother [19]. However, in agreement with previous results from an early experiment in those lines [14], the decrease in body condition at delivery is lower in females from the low line, despite that fact that this line is gestating on average more fetuses.

Immediately after delivery, milk production is low and feed intake is sufficient for covering the nutritional needs for both maintenance and lactation [20]; therefore, body fat reserves are recovered [21]. In accordance with Theilgaard et al. [21], the low and high line increase their body condition between delivery and early lactation; however, the increment is higher in the low line than the high line. A low body condition and high fat mobilization have been related to a high risk of dying or being culled [14,22]; thus, a higher body condition at delivery and a larger fat deposition between delivery from 10 days after delivery in the low line would agree with the lower involuntary elimination rate reported in this line by Argente et al. [11]. In current rabbit production systems, does are mated between 10 and 12 days post-delivery, arriving at the end of weaning with lactation and gestation overlapping [23]. The high energetic needs for milk production and development of fetuses are not entirely compensated with doe’s increasing feed intake at the end of weaning (review by Castellini [24]). Therefore, there is an important increase in the mobilization of dam’s body reserves, which leads them to lose body condition (review by Castellini et al. [25]). In this sense, we also observed a decrease in body condition between 10 days after delivery and weaning in both lines, although the decrease is slightly higher in the low line due to a large number of kits at weaning [18].

We see that selection to reduce residual variance of litter size has a favorable correlated response in body condition and fat mobilization in the dam, playing an important role in coping to environmental challenges.

### 4.2. Effect of Body Condition on Receptivity and Fertility

As previously commented, current rabbit breeding programs are based on an interval between delivery and artificial insemination or mating between 10 and 12 days. Therefore, females have to simultaneously allocate their fatness resources for both maintenance and milk production [26], and poor body condition at mating can limit mating success [27]. Several studies have reported a negative effect of lactation on fertilization rate [20,28]. We stress that our study quantifies for the first time the negative effect of lactating on receptivity and fertility. Non-lactating females have from 10% to 20% more probability to accept to mating than lactating females. The effect is even more relevant for fertility. In this regard, probability of becoming pregnant was from 30% to 35% higher in non-lactating females than in lactating females. No differences in receptivity and fertility were found between lines.

These findings support that lactation mobilizes a large amount of doe’s fat reserves and has an important effect on receptivity and fertility. Therefore, females must arrive to mate with a good body fatness level which will allow them to have a long and successful reproductive lifespan. The low line showed a greater perirenal fat thickness than the high line at mating (8.17 mm versus 7.90 mm). However, this difference was not enough to result in relevant differences between lines in receptivity and fertility.

## 5. Conclusions

Selection for litter size variability showed a correlated response between body condition and fat mobilization. The does selected for litter size homogeneity did better in situations with high-energy demand such as delivery and lactation, compared to those selected for increasing litter size variability. This means the animals in the homogenous line had better health and welfare levels. 

## Figures and Tables

**Table 1:** Features of the marginal posterior distribution of the differences for litter size residual variance at birth (Ve), litter size at first (LS1), second (LS2) and third (LS3) parity, and individual body weight at 4 weeks (IB4w) and at 9 weeks old (IB9w) in rabbits.

	L (*n* = 121)	H (*n* = 124)	D_L-H_	HPD_95%_	*p*
**Ve, kits^2^**	2.78	4.23	−1.45	−2.22, −0.67	1.00
**LS1, kits**	7.54	7.12	0.42	0.26, 1.04	0.90
**LS2, kits**	8.31	7.77	0.54	−0.19, 1.29	0.93
**LS3, kits**	8.93	8.27	0.66	−0.19, 1.48	0.94
**IB4w, g**	732	754	−22	−93.2, 43.9	0.74
**IB9w, g**	1836	1823	13	−70.9, 95.3	0.61

*n*: number of data. L: mean of the low line. H: mean of the high line. D_L-H_: differences between the low and the high line. HPD_95%_: highest posterior density region at 95%. *p*: probability of the difference being >0 when D_L-H_ > 0 or being <0 when D_L-H_ < 0.

**Table 2:** Features of the marginal posterior distribution of the differences for individual body weight (IBW10d) and perirenal fat thickness (PFT10d) at 10 days after delivery for receptivity and fertility.

	Receptive	Non-Receptive	D	HPD_95%_	*p*
IBW10d (g)	3581	3452	129	6.59, 260	0.97
PFT10d (mm)	8.09	7.81	0.28	−0.03, 0.57	0.96
	**Fertile**	**Infertile**			
IBW10d (g)	3593	3511	82	−20, 182	0.94
PFT10d (mm)	8.15	7.87	0.28	0.04, 0.52	0.99

D: differences between receptive and non-receptive does or fertile and unfertile does. HPD_95%_: highest posterior density region at 95%. *p*: probability of the difference being >0 when D > 0 or being <0 when D < 0.
